# DUSP8 phosphatase: structure, functions, expression regulation and the role in human diseases

**DOI:** 10.1186/s13578-019-0329-4

**Published:** 2019-08-27

**Authors:** Tao Ding, Ya Zhou, Runying Long, Chao Chen, Juanjuan Zhao, Panpan Cui, Mengmeng Guo, Guiyou Liang, Lin Xu

**Affiliations:** 1 Special Key Laboratory of Gene Detection and Therapy of Guizhou Province, Zunyi, 563000 Guizhou China; 2 Department of Immunology, Zunyi Medical University, Zunyi, 563000 Guizhou China; 3 Department of Medical Physics, Zunyi Medical University, Zunyi, 563000 Guizhou China; 4 Department of Cardiovascular Surgery, Affiliated Hospital of Guizhou Medical University, Guiyang, 550004 Guizhou China; 5 Department of Cardiovascular Surgery, Affiliated Hospital of Zunyi Medical University, Zunyi, 563000 Guizhou China

**Keywords:** DUSP8, MAPK, Dephosphorylation, Regulation, Diseases

## Abstract

Dual-specificity phosphatases (DUSPs) are a subset of protein tyrosine phosphatases (PTPs), many of which dephosphorylate the residues of phosphor-serine/threonine and phosphor-tyrosine on mitogen-activated protein kinases (MAPKs), and hence are also referred to as MAPK phosphatases (MKPs). Homologue of Vaccinia virus H1 phosphatase gene clone 5 (HVH-5), also known as DUSP8, is a unique member of the DUSPs family of phosphatases. Accumulating evidence has shown that DUSP8 plays an important role in phosphorylation-mediated signal transduction of MAPK signaling ranging from cell oxidative stress response, cell apoptosis and various human diseases. It is generally believed that DUSP8 exhibits significant dephosphorylation activity against JNK, however, with the deepening of research, plenty of new literature reports that DUSP8 also has effective dephosphorylation activity on p38 MAPK and ERKs, successfully affects the transduction of MAPKs pathway, indicating that DUSP8 presents a unknown diversity of DUSPs family on distinct corresponding dephosphorylated substrates in different biological events. Therefore, the in-depth study of DUSP8 not only throws a new light on the multi-biological function of DUSPs, but also is much valuable for the reveal of complex pathobiology of clinical diseases. In this review, we provide a detail overview of DUSP8 phosphatase structure, biological function and expression regulation, as well as its role in related clinical human diseases, which might be help for the understanding of biological function of DUSP8 and the development of prevention, diagnosis and therapeutics in related human diseases.

## Introduction

Protein phosphorylation is a key event that controls cellular responses to external cues [1–3]. Eukaryotic protein phosphorylation most commonly occurs on serine/threonine and tyrosine residues of activated protein kinases [4–9]. Thousands of protein kinases collectively constitute an enormous signaling transduction networks nicely regulating cellular vital movement. In eukaryotic cells, one of the most widely studied signaling pathways is the mitogen-activated protein kinases (MAPKs) pathway which are evolutionally highly conserved and involved in diverse cellular functions, including cellular stress response, proliferation and differentiation [10, 14]. MAPK signaling cascades are made up of a core tier of three kinases: a MAPK kinase kinase (MAP3K) that activates a MAPK kinase (MAP2K), which in turn activates the MAPK by dual phosphorylation of the Thr-X-Tyr activation motif (where X represents any amino acid), in which the upstream kinases are responsible for the phosphorylation and activation of the downstream kinases [11–19]. Extracellular regulated kinase (ERK), c-Jun N-terminal kinase (JNK), and p38 MAPK (p38) are the terminal kinases of the classical MAPK pathways. A wide variety of extracellular stimuli induce phosphorylation and activation of MAPKs including growth factors, inflammatory cytokines, even DNA damage or cellular stress and so forth. However, persistent phosphorylation and activation of MAPK pathways can lead to serious physiological change and pathological injury, even going so far as to induce tumorigenesis. As expected, dephosphorylation mediated by protein phosphatases (PPs) prevents MAPK signaling pathways from being over-activation, which is under compact spatial and temporal control [20–24].

MAPK are switched off by both generic phosphatases and dual-specificity phosphatases (DUSPs) and are further regulated by scaffold proteins, which are usually specific for each of the three major mammalian MAPK pathways [8, 9, 25–27]. Here, a unique phosphatase that dephosphorylates serine, threonine and tyrosine residues and belongs to the largest family of protein tyrosine phosphatases (PTPs). This subfamily is composed of 61 phosphatases and is widely known as the DUSPs because they are capable of dephosphorylating tyrosine and serine/threonine residues in a single substrate [28]. The best-characterized group or classical DUSPs are consisted of 10 MKPs (DUSP1, DUSP2, DUSP4, DUSP5, DUSP6, DUSP7, DUSP8, DUSP9, DUSP10 and DUSP16), which have been identified to dephosphorylate the MAPKs in the activation domain (T-X-Y motif). Based on cellular localization, these DUSPs are subdivided into 3 subsets; nucleus: DUSP1, DUSP2, DUSP4 and DUSP5; cytoplasm: DUSP6, DUSP7 and DUSP9; and both: DUSP8, DUSP10 and DUSP16 [29, 30]. Recently, most typical DUSPs molecules such as DUSP1 [31, 32, 54], DUSP3 [33], DUSP5 [1], and etc. have been known and understood in a systematic, integral and clear way [34–41]. Although, DUSP8, as a emerging phosphatase, involved in the development and progression of multiple human diseases, plays a critical role in negatively regulating the activity via dephosphorylates relevant residues; nevertheless, there is absent of a review to comprehensively summary these findings and its specific significance. Therefore, this review pays focus on the biological structure and function of DUSP8/hVH5, and its relevant regulatory mechanisms, and the relationships in multiple human diseases.

## DUSP8—gene, structure, functions and expression regulation

### Gene and pseudogene

The DUSP8 gene is a protein-coding gene. Human DUSP8 gene, also known as hVH5, is located in human chromosome 11 p15.5 [42, 43]. The mouse DUSP8 gene is called M3/6 and positioned at the distal end of mouse chromosome 7 [44]. Typically, the DUSP8 gene name is universal between human and mouse. In special cases, hVH5 specifically refers to the human DUSP8 gene, while M3/6 is the mouse DUSP8 gene. This gene encodes a 5 kb transcript containing 1.8 kb CDS region that is expressed primarily in the brain, lung and colon and contains translated complex trinucleotide repeats in the coding region [44].

Surprisingly, analyzing DUSP8/hVH-5 transcripts in mammary carcinoma cell lines discovered a sequence with 88% similarity to hVH-5 transcripts. Because this variant of hVH-5 lacked intronic sequences in its genomic structure, so it might be a processed pseudogene of hVH-5 (*ψ*hVH-5). *ψ*hVH-5 transcripts were detected in human peripheral tissues as well as in 11 of 14 breast cancer cell lines. In respect to the normal hVH-5 gene, the pseudogene contains several point mutations and a frame shift due to the deletion of 2 bases that would lead to the truncation of the putative *ψ*hVH-5 product. The intronless pseudogene *ψ*DUSP8/*ψ*hVH-5 contains three different gene sequences; DUSP8P1/DUP8P/DUSP8P3 and DUSP8P2/DUSP8P4 were located on chromosome 10q11.22, and DUSP8P5 are positioned on 11q22.2 rather than hVH-5 presents on chromosome 11p15.5 [45, 46].

### Structure

**Fig. 1:**
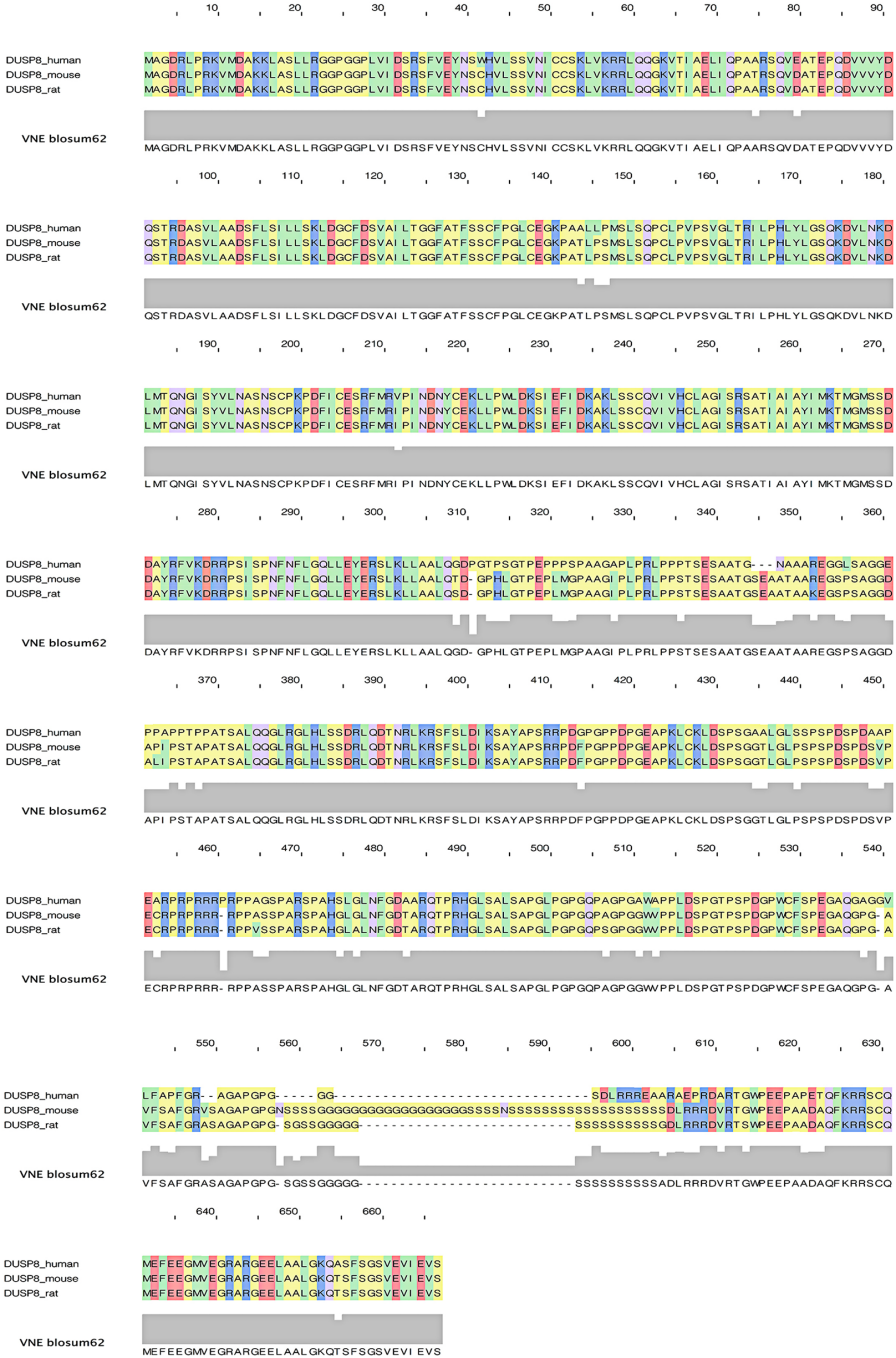
The DUSP8 protein sequence alignment in human, mouse, and rat. The DUSP8 protein sequence of human, mouse, and rat is checked and downloaded from NCBI Database (http://www.ncbi.nlm.nih.gov/protein). And PFAAT Software performed the alignment process and the results are exported from it

**Fig. 2:**
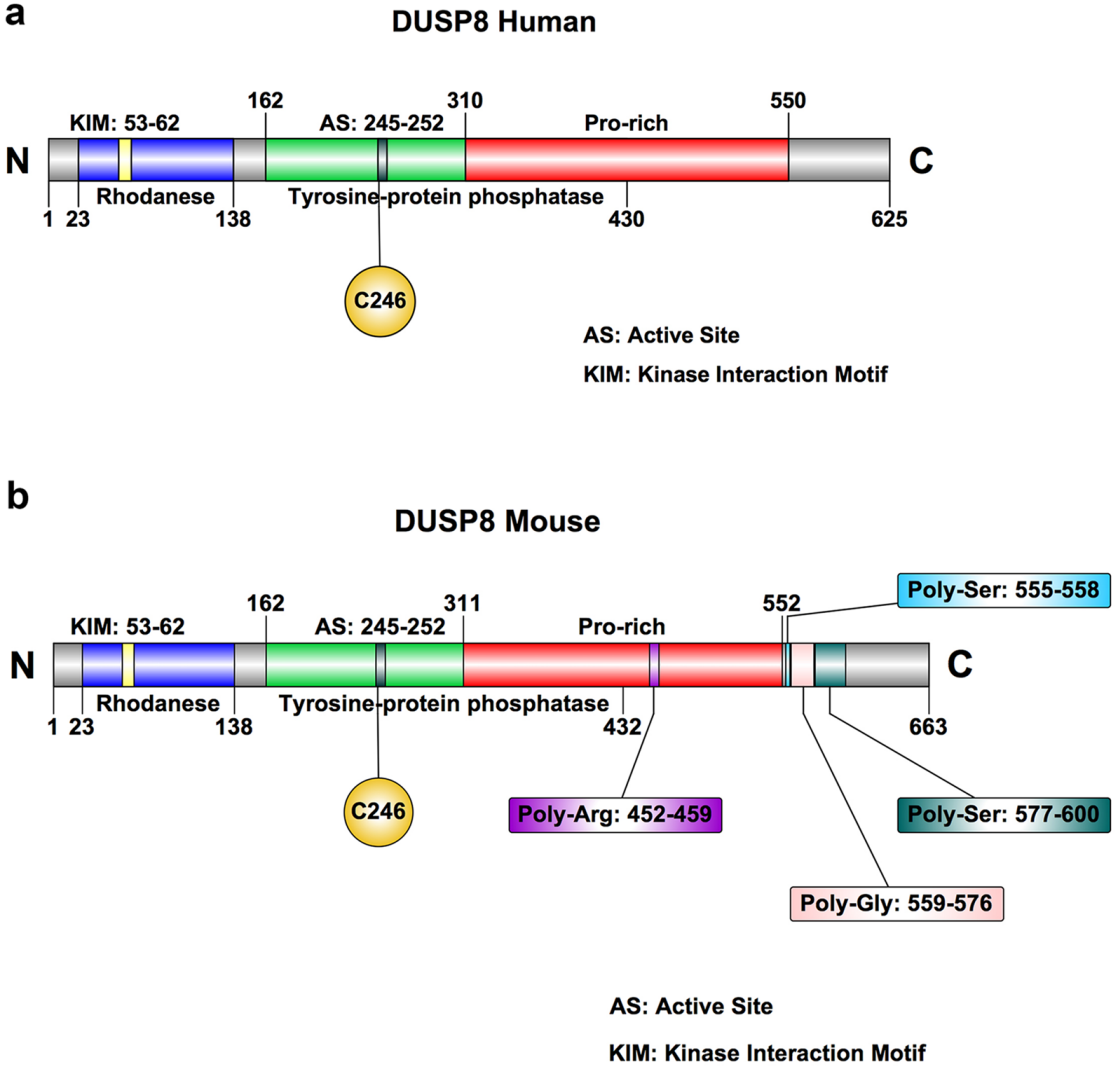
The primary structure of human DUSP8 and mouse DUSP8. The (**a**) human and (**b**) mouse DUSP8 comprise three major domains: Rhodanese domain (in blue), Tyrosine-protein phosphatase domain (in green), and Pro-rich domain (in red). C246 means Cys-246, the catalytic site of DUSP8. *As* active site, *KIM* kinase interaction motif

**Fig. 3:**
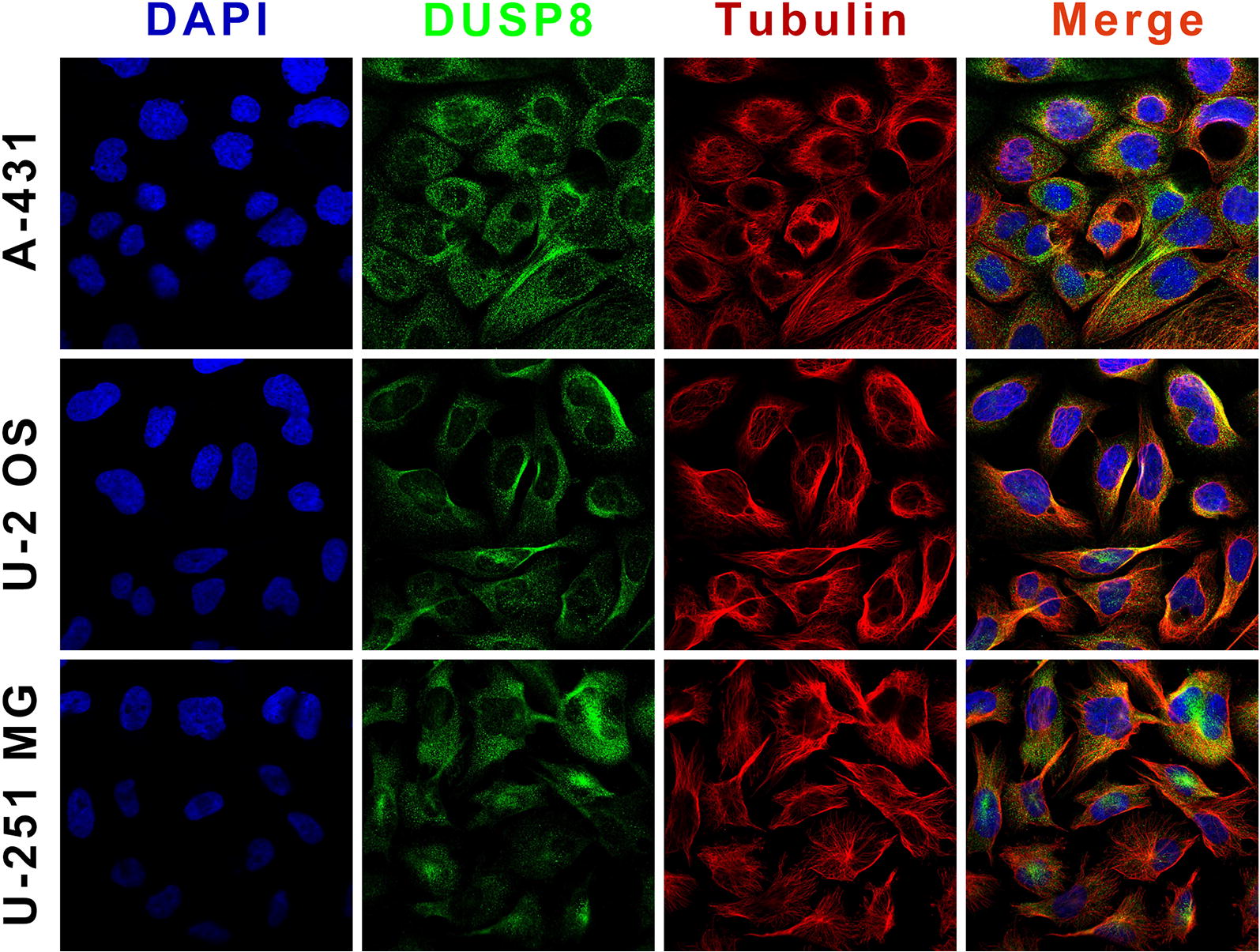
The subcellular location of DUSP8 in A-431, U-2 OS, and U-251 MG cell lines. All images are downloaded from the Human Protein Atals Database (http://www.proteinatlas.org), and visiting the website can acquire more information about protocols, antibodies, and etc. in details

The human DUSP8 gene encodes a protein of 625 amino acids in length, while the mouse DUSP8 protein contains 663 amino acids. Given the complexity of the DUSP8 protein sequence, we align the amino acid sequences of DUSP8 proteins in human, mouse and rat (Fig. 1). To be more specific for human DUSP8, in the primary structure, as shown in Fig. 2a, the protein chain N23-138C and N162-430C encodes Rhodanese domain containing kinase interaction motif (KIM: KLVKRRLQQG, N53-62C) and tyrosine-protein phosphatase domain including active site (AS: HCLAGISR, N245-252C), and has a Pro-rich region (N310-550C) at C-terminal scale. To mouse DUSP8, there are four unique repeated amino acids sequences in Pro-rich region (Poly-Arg: N452-459C, Poly-Ser: N555-558C, Poly-Gly: N559-576C, and Poly-Ser: N577-600C) besides Rhodanese domain and tyrosine-protein phosphatase domain in the same position with human DUSP8 (Fig. 2b). Moreover, according to the Human Protein Atlas Database [47–51], the subcellular localization of DUSP8 is located in both cytoplasm and nucleus of multiple human cells such as A-431 cells, U-2 OS cells, and U-251 MG cells (Fig. 3), and contains a sequence encoding a nuclear translocation signal on protein sequence, but the specific coding sequence remains to be elucidated.

**Fig. 4:**
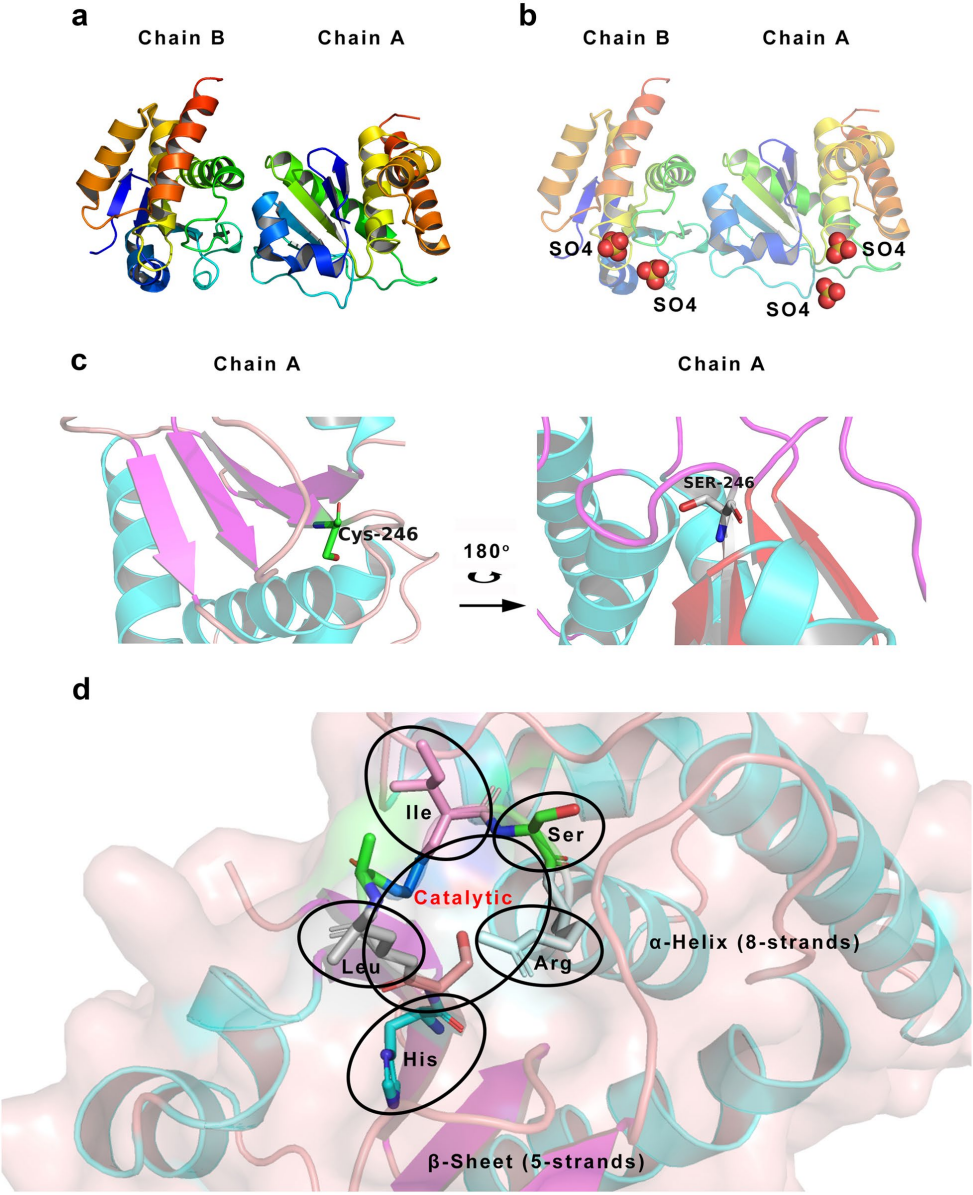
The crystal structure of DUSP8. The PDB file is downloaded from RCSB PDB Database (http://www.rcsb.org). And the file is loaded on Pymol software. **a** The structure of chain A/B is shown. **b** SO_4_ ligand in chain A/B is shown (Red). **c** The catalytic site Cys-246 and **d** the active center (His-245, Cys-246, Leu-247, Ala-248, Gly-249, Ile-250, Ser-251, and Arg-252) in chain A are analyzed and shown

The secondary structure determination of human DUSP8 is identified by protein expression and crystallization using molecular-replacement method with human DUSP10 (PDB: 1ZZW). The final model of DUSP8 at 1.9 Å resolution consists of residues 160–310 of *Chain A*, residues 159–303 of *Chain B* (Fig. 4a), two sulfate ions per chain (Fig. 4b) and 157 water molecules (data not shown), yielding final R_cryst_ and R_free_ values of 17.6 and 20.7%, respectively. Although a three-dimensional structure of DUSP8 has been reported so far, there is a controversy about the catalytic site of DUSP8. The catalytic site of DUSP8 locating in Cys-246 at amino acids sequence of DUSP8 protein is took the place of Ser-246 at PDB database (PDB: 4jmk). For DUSP8, cysteine mutations are necessary to obtain diffraction-quality crystals during structure determination of human DUSP8, possibly because the cysteine residues are susceptible to oxidation. Therefore, the 3D structure and catalytic site containing Ser-246 but not Cys-246 for DUSP8 based on calculation with Pymol is shown in Fig. 4c. Furthermore, the residues including the catalytic site (His-245, Cys-246, Leu-247, Ala-248, Gly-249, Ile-250, Ser-251, and Arg-252) consist of the active site of DUSP8, and these residues like a “pocket” surround the catalytic site, which might be able to interact with T-X-Y motif in distinct MAPKs (Fig. 4d).

### DUSP8 substrates

Unlike DUSP5 has distinct and specific dephosphorylated substrate—extracellular signal-related kinase (ERK) [1], DUSP8 possesses more extensive and broader but highly selective activity of dephosphorylating relevant residues of JNK and p38 MAPK including ERK. For example, DUSP8 as a target molecule of microRNA-21, involved in regulating cell proliferation and collagen synthesis in cardiac fibroblasts via inactivating p38 MAPK and JNK/SAPK signaling [52]. Interestingly, another study about cardiovascular system reported that acute pathologic stress stimulation in hearts upregulated the activation of ERK1/2 in DUSP8 gene knockout mice; in other words, DUSP8 might selectively dephosphorylate the activity of ERK1/2, but not JNK and p38 MAPK, under the circumstance [53]. As for non-MAP kinase, whether DUSP8 exerts dephosphorylated effects on it like DUSP1/MKP1 [54]; or even enhances the activation of MAPKs such as DUSP4 [55], still needs to further study and explore.

### Transcriptional regulation

The expression and activity of DUSP8 can be regulated in a number of ways including gene transcription, protein stability, and phosphatase activity. This multi-level regulation allows for tight control of MAPK activity. Despite DUSP8 is still controversial as an immediate early gene, the expression of DUSP8 mRNA or transcript can be rapidly induced after oxidative stress, heat shock, growth factors, and some small molecular activator such as *O*-tetradecanoylphorbol-13-acetate (TPA/PMA) and anisomycin [56–59]. In K562 human leukemia cells, PMA treatment promptly induced DUSP8 transcripts, and activated the expression of M3/6 (murine DUSP8) completely suppressed PMA-induced phosphorylation of JNK/SAPK [57]. Importantly, the expression of DUSP8 mRNA in adipocytes responded to TNF-α in inflammatory state, which was closely associated to the intensity and constant time of stimulation, and was expressed as MAPK-dependent transcription, suggesting that DUSP8 might be a downstream molecule of MAPK signal transduction and an extremely vital negative regulator in the MAPK signaling pathway rather than DUSP1 and DUSP16 in inflammatory stress [60]. In addition to the unique way of DUSP8 transcription, stimulus-dependent elevation of intracellular Ca^2+^ was defined as excitation-transcription coupling, which affected the transcription of DUSP8 in a cell type specific manner [61]. Finally, as shown in Table 1, 16 candidate transcriptional factors (TFs), such as PAX5, ERα and C/EBPβ and so on, and their putative binding sites on DUSP8 promoter sequence (from transcription start site ahead 2000 bp) are also screened out based on PROMO Database (http://alggen.lsi.upc.es), indicating these TFs might be involved in the transcriptional regulation on DUSP8 expression.

**Table 1:** The potential transcriptional factors binding to DUSP8 promoter

Transcription factors	Numbers of probable binding sites	Representative binding sites (5′–3′)	RE equally	RE query
PAX5	14	CCAGCCC (− 1936 to − 1930 bp)	1.09918	3.98542
		GGGCAGG (− 1688 to − 1682 bp)		
GATA1	1	TATCTG (− 1921 to − 1916 bp)	0.48853	0.20125
ERα	12	TGACC (− 1894 to − 1890 bp)	1.9541	2.1066
		GGTCA (− 1856 to − 1852 bp)		
YY1	18	CCAT (− 1840 to − 1837 bp)	7.81641	6.50071
		ATGG (− 1796 to − 1793 bp)		
GRα	12	CCTGT (− 1957 to – 1953 bp)	3.9082	3.2513
		ACAGG (− 1805 to − 1801 bp)		
p53	5	GGGCAGG (− 1688 to − 1682 bp)	0.24426	0.629
		CCTGCCC (− 1669 to − 1663 bp)		
C/EBPβ	19	GCAA (− 1879 to − 1876 bp)	15.63281	10.04412
		TTGC (− 1615 to − 1612 bp)		
TFII-I	5	CTGTCC (− 1531 to − 1526 bp)	1.46558	1.42439
		GGACAG (− 547 to − 542 bp)		
c-ETS1	2	CTTCCTG (− 1338 to − 1332 bp)	0.12213	0.12024
		CAGGAAG (− 361 to – 355 bp)		
AP-2α	6	GCCTGC (− 1522 to − 1517 bp)	0.48853	1.25819
		GCAGGC (− 1071 to − 1066 bp)		
SP1	1	GCCCCGCCCC (− 931 to − 922 bp)	0.00191	0.02559
c-Myc	1	CACGTG (− 801 to − 796 bp)	0.48853	0.68269
GRβ	1	AATGT (− 403 to − 399 bp)	3.9082	0.95709
RXRα	1	TGAACCC (− 377 to − 371 bp)	0.24426	0.18531
GCF	1	TCCCAGCGC (− 82 to − 74 bp)	0.0916	0.61911
IRF2	1	TCACTT (− 39 to − 34 bp)	0.48853	0.20114

### Post-transcriptional regulation

Due to the long half-life of DUSP8 mRNA, post-transcriptional regulation is also an important part of DUSP8 expression. However, the molecules and related mechanisms involved in the post-transcriptional regulation of DUSP8 remain poorly understood, and more research and studies should be needed for further investigation. Thus, here are listed conserved miRNAs, a class of important gene post-transcriptional factors, that may be involved in post-transcriptional regulation of DUSP8 from TargetScan Database [62, 63] and present literatures.

### Post-translational regulation

As a mitogen-activated protein kinases phosphatase, whether DUSP8 has post-translational modification sites has been widely concerned. Up until now, however, only murine DUSP8, as also known M3/6, has been identified to have phosphorylation sites. It has been shown that M3/6 itself can be phosphorylated by JNK when stimulated with arsenite, but the effect and mechanism of DUSP8 self-phosphorylation has not been studied. Mass spectrometry assay has shown that there are phosphorylation residues *Ser* 515, *Thr* 518 and *Ser* 520 in M3/6, which were JNK-induced phosphorylation sites. And after arsenite stimulation, M3/6 mutated at these sites showed reduced phosphorylation compared to wild-type protein. Interestingly, the expression of the M3/6 phosphorylation mutation delayed the time course of JNK phosphorylation and activation via arsenite stimulation, suggesting that JNK phosphorylation of stress-stimulated M3/6 phosphatase led to a decrease in phosphatase activity and an acceleration of JNK activation [57, 59]. Moreover, to better understand the regulatory function and mechanisms of activation of DUSP8, we predict possible phosphorylation sites of DUSP8 in human and rat by using PhosphoSitePlus^®^ PTM resources from Cell Signaling Technology, Inc. [64] and make a comparison of different phosphorylated residues in DUSP8 between human, mouse, and rat in detail (http://www.phosphosite.org).

## DUSP8—the role in human diseases

### DUSP8 and nervous system

Complex signaling networks including JNK, ERK, p38 MAPK and ERK5 can be activated when the brain exhibits an initial restriction on blood supply and subsequently resumes blood flow. A study has shown that cerebral ischemia and reperfusion in rat hippocampi could induce JNK undergoing complete inactivation following the intense activation, in which after 4 h of reperfusion in rat hippocampi, the activity of DUSP8 was significantly upregulated, accompanied by the dephosphorylation of JNK. The inhibitor of DUSP8, anisomycin, might elevate JNK activity following postischemic reperfusion, indicating that DUSP8 was closely correlated with inactivation of JNK following cerebral ischemia. Innovatively, on one hand, the expression level of heat shock protein (HSP70) increased obviously and was participated in the upregulation of soluble cytoplasmic DUSP8 levels, which induced by cerebral ischemia; on the other hand, inhibition of the activity of HSP70 by using quercetin led to restore the activation of JNK via downregulating the cytoplasmic solubility of DUSP8, suggesting DUSP8 involved in the process of inactivating JNK activation in response to cerebral ischemia required the molecular chaperon HSP70 to impetus the calibration of folding defects [65].

Similarly, Rosiglitazone is a synthetic peroxisome proliferator-activated receptor-γ (PPAR-γ) agonist that prevents cell death following cerebral ischemia in animal models. The study investigated how Rosiglitazone protected neurons from ischemia were conducted in which mice pre-treated with Rosiglitazone were subjected to ischemia for 60 min and then reperfusion. The infarct volume was reduced by Rosiglitazone after ischemia and reperfusion. And PPAR-γ antagonists could reverse the neuro-protective effects. Importantly, Rosiglitazone inhibited a significant increase in the expression of phosphorylated JNK and p38 MAPK in ischemic brain tissue. In addition, Rosiglitazone simultaneously increased the expression of DUSP8 at mRNA and protein level in ischemic brain tissue. Furthermore, knockdown of DUSP8 in primary cultured cortical neurons undergoing oxygen–glucose deprivation diminished the effect of Rosiglitazone on the downregulation of JNK phosphorylation. Therefore, the neuro-protective effect of Rosiglitazone after ischemia was dominantly mediated by upregulation of DUSP8 to block ischemia-induced JNK phosphorylation [66].

For diagnosing biomarker, due to Parkinson’s disease (PD) shares pathological and clinical features with patients diagnosed with progressive supranuclear palsy (PSP) and the differences in disease progression, treatment, and clinical management, it is very urgent need to identify other biomarkers that can distinguish among these diseases. By testing DUSP8 and PTPRC for their diagnostic potential through quantitative PCR assay from 138 blood samples with PD, the results have shown that compared to PD patients and healthy controls, the relative abundance of PTRPC mRNA significantly decreased in PSP patients, whereas there was no obvious difference in the expression level of DUSP8, suggesting DUSP8 might not be suitable to become a biomarker for assisting to diagnose PD or PSP [67].

As a novel member of immediate early genes that encode enzymes of MKP family, DUSP8 also plays a unique role in drug addiction. In the nucleus accumbens ŽNAc., caudate putamen, frontal cortex and hippocampus of rat brain, i.p. injection of cocaine, amphetamine and caffeine induced DUSP8 mRNA expression within 40 min, with a maximal affect in the NAc. Compared with c-Fos and EGR-1 immediate early genes, the expression of DUSP8 mRNA in the NAc and hippocampus was obvious upregulation and continuous after cocaine injection for 10 days as a single injection later. This study demonstrated from another perspective that DUSP8 acted as a negative regulator of MAPK and was involved in MAPK-mediated central phschostimulants addiction [68].

### DUSP8 and circulation system

High glucose could promote the proliferation and collagen synthesis of rat cardiac fibroblasts, accompanied by the upregulated expression of micoRNA-21, a well-known molecule of extensive studies on multiple diseases, suggested the critical role in diabetic cardiomyopathy. DUSP8, a direct target of miR-21 binding 3′-UTR of DUSP8 inhibited the expression at post-transcriptional level, was involved in regulating cell proliferation and collagen synthesis in cardiac fibroblast via p38 MAPK and JNK/SAPK signaling pathway [52].

Furthermore, a study has documented that DUSP8 gene deletion (knockout) mice exhibited baseline concentric heart remodeling, which was enhanced during stress stimulation. This concentric ventricular remodeling was associated with increased myocardial contractility at baseline and prevention from dilation and heart failure in two different induced pathology models. In addition, loss of DUSP8 resulted in increasing ERK1/2 activation at baseline and after acute pathological stimulation, whereas p38 and JNK kinase were unaffected. In contrast, overexpression of DUSP8 led to decreased phosphorylation of all MAPKs studied, ventricular dilatation and greater propensity for heart failure. This study suggested that DUSP8 regulated the dynamics of cardiac MAPKs signaling pathway, which directly affected ventricular remodeling and heart failure propensity [53].

### DUSP8 and urinary system

Renal branching morphogenesis is a necessary process to mammalian kidney development, which is defined as growth and branching of the ureteric bud (UB) and its derivatives. And the formation of the collecting ducts and pelvis of mature kidney also needs UB to grow, branch, and differentiate. Defects with renal branching morphogenesis result in congenital renal dysontogenesis, characterized by aberrant collecting system morphology and low nephron number. Integrin-linked kinase (ILK) is definitely an intracellular scaffolding protein with essential cell specific functions in the embryonic and mature mammalian kidney. Based on whole genome expression analysis of whole kidney mRNA in transgenetic mice with ILK loss in the ureteric cell lineage, the expression of six genes relevant kidney maturity including Wnt11 were downregulated, whereas the expression of DUSP8 increased in ureteric tip cells, accompanying with the downregulation of phosphorylated p38 MAPK in kidney tissues with ILK deficiency. Moreover, overexpression of DUSP8 in murine inner medullary collecting duct 3 (mIMCD3) cells significantly suppressed the activation of p38 MAPK. Furthermore, overexpression of DUSP8 mediated by adenovirus also inhibited ureteric branching and the activity of p38 MAPK. This study vests DUSP8 a brand new function of kidney differentiation and maturation [69].

### DUSP8 and immune system

The proliferation, differentiation, secretion of cytokines, and execution of immune responses of various types of immune cells are inseparable from the strict and orderly regulation of activation and deactivation of MAPK pathway. BAF3 cells were precursor B cells that undergone apoptosis following IL-3 withdrawal or ceramide treatment. JNK/SAPK in BAF3 cells was stimulated by ceramide and responded to IL-3 stimulation during cell proliferation. Expression of DUSP8 in BAF3 cells prevented ceramide from stimulating JNK/SAPK activity. The proliferation of BAF3 cells expressing DUSP8 with IL-3-stimulated was inhibited [70]. In addition, DUSP8 not only played an immune response-suppressing gene to regulate responses to between host and Aspergillus fumigatus and Candida albicans, but also involved in suppressing Ras pathway of T cell signal transduction in renal transplant [71]. However, the exact roles of DUSP8 in immune system and immune response remain to be fully elucidated [72, 73].

### DUSP8 and cancer

For human colorectal carcinoma (CRC), our previous work observed the effect of antisense oligonucleotides (ASOs) against miR-21 on the growth and metastasis of CRC in vivo using a xenograft model of human CRC. We found that ASOs could effectively inhibit the growth and metastasis of CRC in vivo, accompanied by downregulated expression of miR-21 and reduced transduction of the AKT and ERK pathway. Mechanically, global gene expression analysis showed that the expression of DUSP8, a target of miR-21, was upregulated in tumor mass. Furthermore, overexpression of DUSP8 could remarkably suppress the proliferation and migration of CRC cells in vitro. Finally, downregulation of DUSP8 could abrogate the effects of ASOs against miR-21 on the proliferation and migration of CRC cells, as well as altered transduction of the AKT and ERK signaling pathway. This is the first time we demonstrated that the DUSP8 mediated pathway plays a pivotal anti-cancer role on ASOs against miR-21 inhibiting the growth and metastasis of CRC [74].

In contrast, other study in prostate cancer reported that Phenyl isocyanate (PEITC) was capable of inducing activation of JNK and apoptosis in prostate cancer cell lines with different p53 status. The JNK dephosphorylation and deactivation rate of cells exposed to PETIC was reduced by PEITC directly downregulating the expression level of DUSP8 via proteasome-dependent mechanism, suggesting that PEITC targeting DUSP8, similar as a oncogene in prostate cancer, could restore activity of JNK through inhibiting the expression and activation of DUSP8 [75]. Therefore, these studies suggested that the biological role of DUSP8, waiting for deep investigation, in the development of cancers is complex.

### DUSP8 and genetics

In type II diabetes, based on the pyrosequencing of DNA methylation, one study investigating 40 single nucleotide polymorphisms (SNPs) previously associated with type II diabetes a CpG site (introduce or remove), and examining whether these CpG-SNPs were correlated with differential DNA promoter methylation in pancreatic islets of 84 human donors, has revealed that 19 of 40 (48%) type II diabetes-related SNPs with introducing or removing a CpG site. Then, DNA methylation data were successfully generated 16 genes including DUSP8 (SNP ID: rs2334499) from 19 CpG-SNP loci, which had 4 linkage-disequilibrium (LD) SNPs. Combining these 19 type II diabetes-related CpG-SNPs presented strong LD with a total of 295 SNPs including 91 CpG-SNPs [76].

Moreover, another study has shown that evidence of genetic linkage of alcohol dependence was found on chromosome 11p15.5 in autosomal scans of aboriginal populations in Southwestern America. And DUSP8 gene not only met the candidate criteria, but also played a crucial role in various pathways known to constitute pathophysiological mechanisms leading to the development of alcohol dependence, which was sequenced to identify polymorphisms that might be associated with disease. The results revealed that 4 patent polymorphisms were characterized in DUSP8, one of which resulted in an amino acid substitution (DUSP8 C712T). However, there was not significant association between DUSP8 C712T polymorphism and alcohol dependence by using genotyping of analyzing functional of DUSP8 in 463 Southwestern Native Americans [77].

## Conclusion

**Fig. 5:**
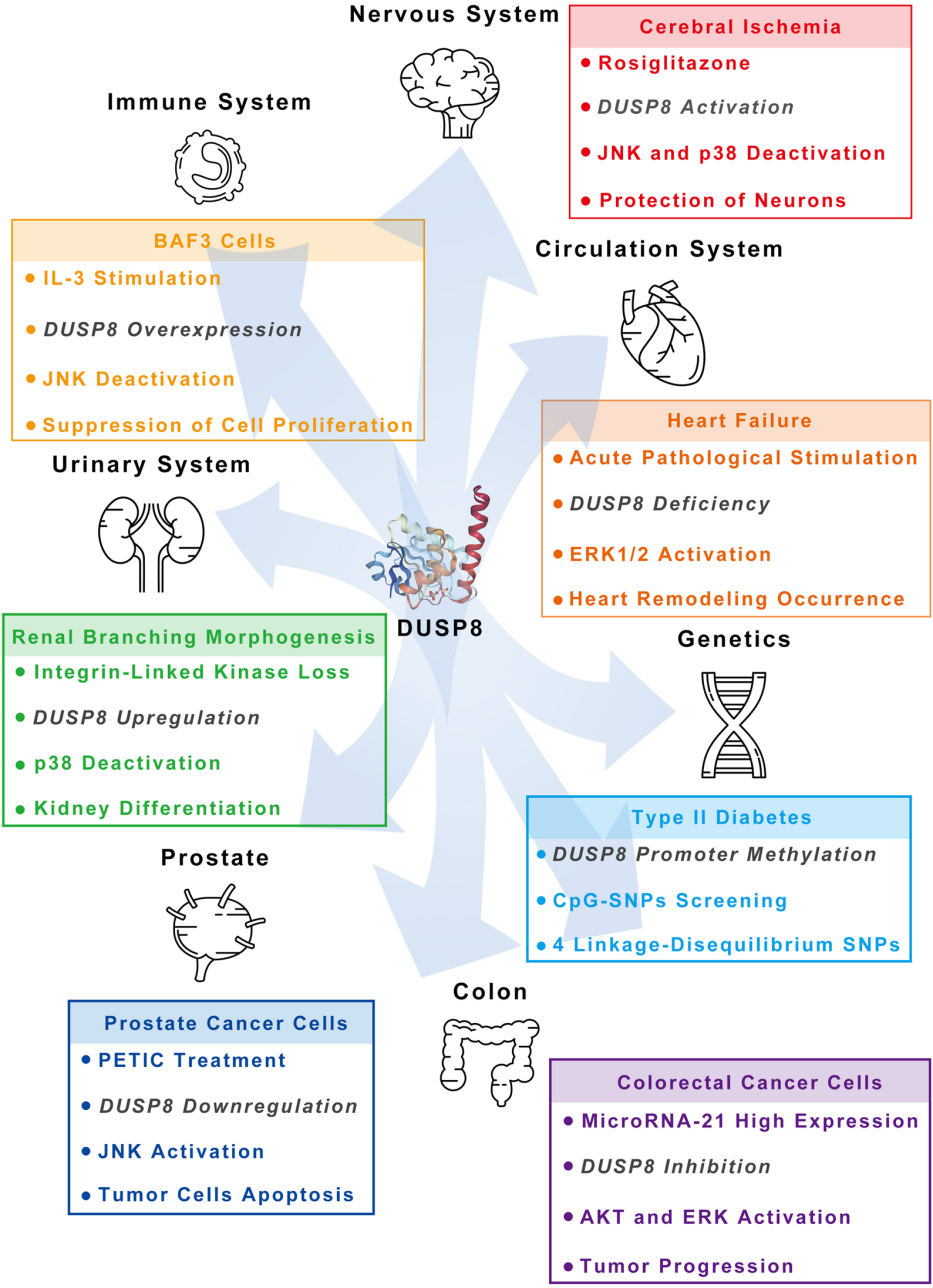
The schematic diagram of the function of DUSP8 in main organs. The physiological and pathological function of DUSP8 involved in the development or disease of seven main organs are shown as separate items for clarity of the description in the main text

Accumulating evidence has shown that DUSP8, a unique member of DUSPs family, is emerging as a critical negative regulator for MAPKs pathway and involved in cell oxidative stress response and cell apoptosis, as well as the development of various human diseases. In the future, there still are some core issues, mainly from three aspects, should been further addressed. The first is the regulation mechanism of DUSP8 expression in multiple types of cells and tissues. Even there have been some mechanisms of DUSP8 expression have been investigated as above description. However, there are still some problems need to be illustrated, such as what is the core sequence of promoter of DUSP8 and its related transcription factors or elements in various cells and tissues; what is the mechanism on the protein stability of DUSP8, specially the degradation of endogenous DUSP8 protein, and so on… The second is the biological structure of DUSP8. Due to the unknown crystal structure of the DUSP8 KIM motif in Rhodanese domain, the analysis and clarification of the recognition mechanism of DUSP8 on different MAPKs substrates would be further conducted. Especially, as a result of DUSP8 itself has multiple potential phosphorylation sites, it is unclear yet whether DUSP8 exerts dephosphorylation function depends on the phosphorylation of its specific sites or whether phosphorylation of different sites is related to the substrate specificity of dephosphorylation. Meanwhile, the origin of nuclear translocation signal on DUSP8 protein sequence and the subcellular co-location of both DUSP8 and its substrates including classical MAPKs such as ERK, JNK and p38 MAPK or other factors also need to be identified. Third, the exact role of DUSP8, including *ψ*hVH-5, in the development of various human diseases, is still unknown. DUSP8 is widely involved in the development of multiple clinical diseases (Fig. 5). However, the underlying connection of DUSP8 and MAPKs signaling pathway involved in the development and therapeutics of clinical diseases remains to be further clarified.

In all, successive research works on the exploration on more exact regulation expression mechanism and biological structure of DUSP8, as well as the connection between DUSP8 and clinical diseases, will undoubtedly provide a better prospect of acknowledgement on DUSPs family member including DUSP8 and the clinical application for DSUPs-based prevention, diagnosis and therapeutic strategies in clinical diseases.

## Data Availability

Not applicable.
